# High prevalence of low bone mineral density in patients within 10 years of onset of ankylosing spondylitis: a systematic review

**DOI:** 10.1007/s10067-012-2018-0

**Published:** 2012-06-16

**Authors:** M. A. C. van der Weijden, T. A. M. Claushuis, T. Nazari, W. F. Lems, B. A. C. Dijkmans, I. E. van der Horst-Bruinsma

**Affiliations:** 1 Department of Rheumatology, VU University Medical Center, Room 3A-64, P.O. Box 7057, 1007 MB Amsterdam, The Netherlands; 2 Reade, Department of Rheumatology, Amsterdam, The Netherlands

**Keywords:** Ankylosing spondylitis, Bone mineral density, Osteopenia, Osteoporosis, Vertebral fractures

## Abstract

Ankylosing spondylitis (AS) is a chronic inflammatory rheumatic disease. Decreased bone mineral density (BMD) is a common complication of AS, with a prevalence range of 19 to 62 %. Many studies have shown decreased BMD in AS with long disease duration, but only a few studies investigated BMD in early AS. The prevalence of decreased BMD in early disease stages of AS has not yet been clearly described, and for that reason, we reviewed the literature which describes the prevalence of decreased BMD in AS patients with a short disease duration (<10 years). In this review, we included articles which used the modified New York criteria for the diagnosis of AS, included patients with a disease duration of less than 10 years, and used the WHO criteria for osteopenia and osteoporosis. Decreased BMD was defined as a *T* score < −1.0, including both osteopenia and osteoporosis. For this review, only articles that acquired BMD data of lumbar spine and femoral neck by DXA were used. The literature search provided us 35 articles of which 7 matched all our criteria, and they will be further outlined in this review. The overall prevalence of decreased BMD of the articles reviewed is 54 % (*n* = 229/424) for lumbar spine and 51 % (*n* = 224/443) for femoral neck. The prevalence of osteopenia vs. osteoporosis for lumbar spine is 39 vs. 16 % and for femoral neck, 38 vs. 13 %. This review showed a high total prevalence of 51–54 % decreased BMD and 13–16 % osteoporosis in AS with a short disease duration. This high prevalence was not to be expected in a relatively young and predominantly male population. Further research is needed to determine the clinical relevance of this low BMD by investigating the relation between low BMD and vertebral and nonvertebral fractures at this early stage in AS.

## Introduction

Ankylosing spondylitis (AS) is a chronic inflammatory rheumatic disease of the axial skeleton, which is characterized by low back pain and stiffness for more than 3 months that improves with exercise, but is not relieved by rest. Other important symptoms are restriction of motion of lumbar spine and limitation of chest expansion. Typical radiological features of AS are sacroiliitis on X-ray and bridging syndesmophytes of the spine, which usually take many years to develop, and besides that, not all AS patients develop these syndesmophytes. The first symptoms usually arise at an age younger than 30 years. Men are more often affected than women, and 90–95 % of the AS patients are HLA-B27 positive. The prevalence of AS ranges between 0.1 and 1.4 % in Europe and is partly dependent on the prevalence of HLA-B27, which differs in populations with different ethnic backgrounds [1–3].

Osteoporosis in terms of decreased bone mineral density (BMD) is a common complication in AS patients. The prevalence ranges from 19 to 62 % [4–6]. The decrease of BMD can be found both in the hip as well as in the spine and depends on disease duration and presence of syndesmophytes of the spine [7, 8]. Besides osteoporosis, also vertebral fractures are an important complication of long-standing AS. The prevalence described of known vertebral fractures ranges between 1 and 19 %; however, usually most of them remain unrecognized [4, 9–12]. In many cases, neither the patient nor the physician is aware of this increased fracture risk among the relatively young and predominantly male AS population which can result in delay of diagnosis and complications.

Osteoporosis in AS with a long disease duration has been well known for quite some time now [9, 13–17]. Currently, the treatment options for AS have improved since the use of TNF-blocking agents, and the diagnosis is made at an earlier stage of the disease. The knowledge of the prevalence of decreased BMD in this early group, however, is limited despite the fact that a decrease of BMD increases the fracture risk [8, 10, 18, 19]. For this reason, we decided to review the literature in order to investigate the prevalence of decreased BMD in patients with AS with a relatively short disease duration (<10 years). Secondly, we focused at the clinical relevance of low BMD in this early population, especially in relation with the risk of vertebral fractures, since vertebral fractures are a major and serious cause of morbidity in long-standing AS patients.

## Methods

All patients included in the studies we used for this review had to fulfill the diagnosis of ankylosing spondylitis according to the modified New York criteria [2]. BMD was defined according to the established criteria of the World Health Organization (WHO), which is based on data of postmenopausal women as no criteria of osteopenia and osteoporosis are available for males. Normal bone density was defined as *T* score ≥ −1.0, osteopenia as −2.5 < *T* score < −1.0, and osteoporosis as *T* score ≤ −2.5 [20, 21]. The *T* score corresponds to the number of standard deviations (SD) from any result of the peak bone mass. We defined “decreased or low BMD” in this review as *T* score ≤ −1.0 SD, including both osteopenia and osteoporosis.

For this review, disease duration was defined as “time since diagnosis” according to the modified New York criteria [2]. However, in most articles, the definition of disease duration was not available or not clearly described, and when described, in most cases “time since diagnosis” was used as disease duration. Therefore, we decided that disease duration (defined as “time since diagnosis”) of less than 10 years was the inclusion criterion for our search, and not symptom duration (defined as “time since first symptoms”).

The diagnosis of AS, disease duration of less than 10 years, and the outcome BMD with prevalence numbers were our main inclusion criteria. For this review, we only used BMD data of the articles that were acquired by dual X-ray absorptiometry (DXA), and all other measurements performed were left out. Furthermore, we only used measurements of the lumbar spine and femoral neck. Other measurements will be briefly mentioned.

The databases used were PubMed, MEDLINE, Embase, and Google Scholar with the following free search terms: “ankylosing spondylitis,” “osteoporosis,” and “bone mineral density.” The search in PubMed was also performed with these free terms as MeSH terms. In our search, we always used the Boolean operator “AND.” Through Web of Science, we looked at which other articles had quoted our main articles. To expand our search even more, we also used the references mentioned by the articles and reviews we found. Case reports were excluded because they have primarily a signaling function, and extrapolation from their results is not valid.

### Statistics

An analysis was made of the overall prevalence of decreased BMD and for the particular location lumbar spine or femoral neck. This was done by adding up the number of patients having a decreased BMD at that particular location. Subsequently, the total number of patients found with decreased BMD was divided by the total number of patients involved in those articles.

## Results

### Search results

A literature search was conducted using the databases and search terms mentioned above. This global search provided us with 35 articles [4, 7–10, 13, 14, 16–19, 22–45]. Using the mentioned inclusion and exclusion criteria, 11 articles were excluded because of an unknown disease duration or disease durations longer than 10 years [9, 14, 16, 17, 34–39, 44]. Another 16 articles were left out because the prevalence number was not described [5, 8, 10, 13, 18, 23, 25, 28–33, 40–42]. One article was excluded because it was a case report [43]. One study investigated early SpA patients, but the subgroup of early AS was clearly described and therefore also included in this review [19]. This resulted in seven articles which matched all our criteria, and they will be further outlined below (Table 1).

**Table 1:** Prevalence of low bone mineral density of lumbar spine and femoral neck

Authors	Number of patients	Country	Male gender	Mean age (years)	Mean disease duration (years)	Prevalence low BMD of lumbar spine	Prevalence osteopenia/osteoporosis lumbar spine	Prevalence low BMD of femoral neck	Prevalence osteopenia/osteoporosis femoral neck
Aydin et al. [24]	*n* = 58	Turkey	100 % (*n* = 58)	38.2	9.3 ± 6.5	ND	ND	59 % (*n* = 34)	43 % (*n* = 25)/16 % (*n* = 9)
Baek et al. [27]	*n* = 76	Korea	100 % (*n* = 76)	28.1 ± 7.9	9.4 ± 5.1	50 % (*n* = 38)	42 % (*n* = 32)/8 % (*n* = 6)	16 % (*n* = 12)	14 % (*n* = 11)/1 % (*n* = 1)
Dos Santos et al. [22]	*n* = 39	France	100 % (*n* = 39)	37.6 ± 9.1	8.4 ±6.3	46 % (*n* = 18)	ND	ND	ND
Karberg et al. [7]	*n* = 27 (group1)	Germany	63 % (*n* = 17)	34.2 ± 11.8	2.5 ± 1.1	56 % (*n* = 15)	41 % (*n* = 11)/15 % (*n* = 4)	67 % (*n* = 18)	56 % (*n* = 15)/11 % (*n* = 3)
	*n* = 48 (group2)	Germany	65 % (*n* = 31)	38.1 ± 11.8	7.0 ± 1.8	46 % (*n* = 22)	31 % (*n* = 15)/15 % (*n* = 7)	75 % (*n* = 36)	50 % (*n* = 24)/25 % (*n* = 12)
Kim et al. [26]	*n* = 60	Korea	85 % (*n* = 51)	32.1 ± 1.2	5.5 ± 0.9	57 % (*n* = 34)	37 % (*n* = 22)/20 % (*n* = 12)	73 % (*n* = 44)	42 % (*n* = 25)/33 % (*n* = 20)
van der Weijden et al. [19]	*n* = 94	The Netherlands	73 % (*n* = 69)	37.2 ± 9.4	7.9 ± 6.2	46 % (*n* = 43)	37 % (*n* = 35)/9 % (*n* = 8)	34 % (*n* = 32)	31 % (*n* = 29)/3 % (*n* = 3)
Vasdev et al. [45]	*n* = 80	India	100 % (*n* = 80)	32.9 ± 8.3	8.1 ± 5.8	74 % (*n* = 59)	45 % (*n* = 36)/29 % (*n* = 23)	60 % (*n* = 48)	49 % (*n* = 39)/11 % (*n* = 9)
Total	*n* = 482		87 % (*n* = 421/482)	34.5	7.7	54 % (*n* = 229/424)	39 % (*n* = 151/385)/16 % (*n* = 60/385)	51 % (*n* = 224/443)	38 % (*n* = 168/443)/13 % (*n* = 57/443)

*n* number of patients, *ND* not done

### Population

Karberg et al. recruited 103 German AS patients [7]. Dependent on disease duration, they divided their patients into three groups—group 1, patients with disease duration of <5 years (*n* = 27); group 2, patients with disease duration of 5–10 years (*n* = 48); and group 3, patients with disease duration >10 years (*n* = 28). For our review, we excluded group 3 because of a disease duration of more than 10 years. The study group of Aydin et al. contained 58 Turkish male patients with AS [24]. Two studies were performed in Korea: a study by Baek et al. which included 76 Korean men with AS and a study by Kim et al. which included 60 AS patients [26, 27]. Thirty-nine French white, male patients with AS were included in the study of Dos Santos et al. [22]. In the study of Vasdev et al., 80 Indian male AS patients were studied [45]. The last study included in this review derived from our group by van der Weijden et al. contains 94 Dutch early AS patients [19].

### Detection techniques

All the studies included in this review used DXA to measure BMD. Karberg et al. also used dual-energy quantitative computed tomography and peripheral quantitative computed tomography and compared these results with DXA [7]. Aydin et al. used DXA to measure BMD in the left proximal femur (femoral neck, trochanter area, and total hip), and all scans were analyzed by the same investigator [24]. BMD in the proximal femur was also measured by Baek et al. [27]. They also measured BMD of the lumbar spine (L2–L4). Kim et al. determined BMD at similar places, lumbar spine (L1–L4) and femoral neck [26]. Karberg et al. performed also the same measurements; they measured lumbar spine (L2–L4) and femoral neck, just like van der Weijden et al. [7, 19]. Dos Santos et al. used DXA to measure whole body BMD and regional BMD of the head, arms, whole spine, lumbar spine, pelvis, and legs [22].

### Outcome

The majority of the 482 patients described in the seven studies were male (87 %), with a mean age of 34.5 years and a mean disease duration of 7.7 years (Table 1). The mean prevalence of low BMD in the early stage of AS was 51 % (range, 16–75 %) for the femoral neck and 54 % (range, 46–74 %) for the lumbar spine. The mean prevalence of osteopenia for the femoral neck was 38 % (range, 14–56 %) and 39 % (range, 31–45 %) for the lumbar spine. Osteoporosis was described in 13 % (3–25 %) of the patients at the region of the femoral neck and in 16 % (range, 8–29 %) of the lumbar spine.

Additional numbers were presented by Aydin et al., who also reported low BMD in 57 % (*n* = 33/58) of the cases of trochanter and 55 % (*n* = 32/58) of total hip [24]. Bone loss was found in 40 patients (69 %) in at least one of three regions (femoral neck, trochanter, and/or total hip). Overall, 22 % (*n* = 13) had osteoporosis and 47 % (*n* = 27) osteopenia using the WHO criteria for osteoporosis. A lower frequency of decreased BMD of femoral neck and trochanter was found by Baek et al. [27]. The frequency they found was 16 % (*n* = 12/76) in femoral neck and 8 % (*n* = 6/76) in trochanter. Vasdev et al. also studied BMD in femur trochanter and Ward’s triangle [45]. In the femur trochanter, they found 50 % osteopenia and 5 % osteoporosis, and in Ward’s triangle, 31 % osteopenia and 2 % osteoporosis.

### Description of risk factors of low BMD

Decreased BMD can occur due to several factors of which old age and female gender are the most important ones. However in early AS, most patients are younger than 40 years of age, and therefore, high age does not seem to be the most important risk factor in this group.

The studies described here dealt differently with the other risk factors of low BMD. Four out of the seven articles excluded patients with common risk factors for osteoporosis, such as use of glucocorticosteroids, hip fractures in the family, and immobilization [22, 24, 27]. Aydin et al. also excluded patients using NSAIDs and DMARDs in the last month, as well as different types of secondary diseases that could cause decreased BMD (i.e., endocrine or metabolic diseases, liver or renal diseases, hip fractures in family, and extremity fractures in the patient). Vasdev et al. and Dos Santos et al. [22, 45] also excluded patients with secondary diseases with the latter also excluding patients with alcohol and tobacco abuse. The remaining studies used no exclusion criteria for their patient selection [7, 19, 26].

The presence of syndesmophytes in patients included in the articles reviewed could be important since syndesmophytes might falsely increase BMD measured by DXA. Dos Santos et al. therefore excluded patients with syndesmophytes [22]. The others described the percentage of syndesmophytes. Karberg et al. found 26 % syndesmophytes in group 1 (*n* = 7) and 55 % in group 2 (*n* = 26), whereas Baek et al. found 36 % syndesmophytes (*n* = 27) [7, 27]. Vasdev et al. reported syndesmophyte formation in 23 % [45] of their patients. Van der Weijden et al. mentioned in their article that only very few patients had a small syndesmophyte which probably could not have influenced their presented results [19]. Aydin et al. and Kim et al. did not mention anything about the presence of syndesmophytes [24, 26].

Another risk factor for osteoporosis is the postmenopausal state of women. Kim et al., Karberg et al., and van der Weijden et al. were the only ones who included women in their studies [7, 19, 26]. The number of women in these studies was low, 15 % (*n* = 9, Kim et al.), 37 % (*n* = 10, Karberg et al. group 1), 35 % (*n* = 17, Karberg et al. group 2), and 27 % (*n* = 25, van der Weijden et al.). In the first two studies, menopause was not further pointed out in relation to the reported percentage of decreased bone density. In the study of van der Weijden et al., only two women were postmenopausal, one of them with low BMD and the other with normal BMD. Therefore, female gender does not explain the high prevalence of low BMD in the 482 patients reported in this review.

## Discussion

The literature search for the prevalence of decreased BMD and osteoporosis in AS within the first decade after diagnosis revealed a high mean prevalence of decreased BMD between 51 % of the femoral neck and 54 % of the lumbar spine. Moreover, the prevalence of osteoporosis was 16 % for lumbar spine and 13 % for femoral neck. This corresponds with prevalence numbers found in long-standing AS patients. The variation in prevalence in the studies reviewed is probably mainly caused by differences in the presence or exclusion of cases with risk factors for low BMD between the included patients and the heterogeneity of the populations. However, the fact that the various studies performed within different populations show an almost equally raised prevalence of low BMD strengthens the conclusion that high prevalence of low BMD is already present at the early stages of AS. Obviously, this might be clinically relevant since low BMD is related to an elevated fracture risk in primary osteoporosis in postmenopausal women and men. In line with that, Cooper et al. and Vosse et al. have shown that vertebral fracture risk is elevated in patients with AS [31, 46].

Some limitations of this review must be mentioned. Our choice of BMD outcome resulted in exclusion of several articles. We choose to only include studies which gave WHO defined *T* scores as well as a prevalence to be able to correctly compare and summon BMD outcomes. Also, for decreased BMD, we used the threshold of a *T* score < −1.0, as defined by the WHO. In ISCD 2007, decreased BMD for males younger than 50 years was described as a *T* score < −2.0. However, as all studies used osteopenia (−2.5 < *T* score < −1.0) or osteoporosis (*T* score ≤ −2.5) as BMD outcome measure, we used the osteopenia cutoff point as “low BMD.”

The seven studies used for this systematic review were conducted in different countries—Korea [26, 27], France [22], Germany [7], Turkey [24], India [45], and The Netherlands [19]. These countries differ in background risks of osteoporosis due to racial differences, dietary habits, and sun exposure. The differences in prevalence of decreased BMD between areas in a healthy population were shown by Lunt et al. They found that BMD of the spine appears to vary in 16 European populations, from 0.934 to 1.0670 g/cm^2^ in women and from 1.047 to 1.262 g/cm^2^ in men [47–49]. Furthermore, the background prevalence of AS differs among several countries, roughly correlating with the prevalence of HLA-B27 [1, 3]. Beside the genetic factors predisposing to AS, the prevalence of decreased BMD in (early) AS is also influenced by differences in environmental (risk) factors among countries, i.e., vitamin D deficiency, smoking, low body mass index, use of corticosteroids, and anti-TNF alpha therapy [50–54]. Unfortunately, four of the studies reviewed excluded patients with common risk factors for osteoporosis, while the others did not. Besides that, the average participant number of the studies presented was 69 patients which is a relatively small number to extrapolate to a population prevalence [7, 19, 22, 24, 26, 27, 45].

Next to general risk factors for low BMD, also disease-specific risk factors like high disease activity and inflammation are of influence on BMD [9, 13, 55]. Some authors suggest that inflammation can explain the major part of the etiology of decreased BMD with cytokines as interleukin-1 and TNF alpha as primary mediators [18, 23, 56–58], stating that low BMD in AS patients with inflammatory back pain is related to inflammation, whereas Aydin et al. as well as others found that bone loss was related to low serum sex hormone levels in AS [14, 24]. However, Bronson et al. were unable to find a correlation between testosterone levels and decreased BMD [15]. Remarkably, Karberg et al. found that more patients with syndesmophytes had low BMD than those without and therefore suggested that bone loss and bone growth occur parallel, also in early stages of AS [7]. This could not be confirmed by the study of van der Weijden et al. because very few syndesmophytes were found in the early stage of disease in combination with a high prevalence of low BMD (46 %) [19]. In this study, high disease severity indicated by impaired physical function (high BASMI and BASFI levels) and high CRP levels appeared to be strongly associated with low BMD AS within 8 years of onset. So, also in early AS, there seems to be an important role for inflammation [19, 58].

A point worth mentioning regarding this review is the definition of disease duration. In this review, disease duration was defined as the “time since diagnosis.” Aydin et al. and van der Weijden et al. both mentioned in their articles the duration from the “moment of diagnosis” and the duration from the “moment of first symptoms” (both <10 years) [19, 24]. Karberg et al., however, defined disease duration as the “time since first symptoms” (also <10 years) [7]. Because the definition of Karberg et al. would only have shortened the disease duration (the mean delay between onset of first symptoms and diagnosis can run up to 8–10 years), this study was considered to be eligible for this review [59–61]. And above that, the results from this study showed already a high prevalence of decreased BMD, which only indicates more that decreased BMD is already present in the (very) early stages of AS.

Another point worth mentioning are the difficulties of diagnosing osteoporosis in AS studies in general. This problem is due to the fact that DXA of lumbar spine has a low sensitivity in detecting decreased BMD in AS patients with a long disease duration due to occurrence of bridging syndesmophytes and ligamentous ossification that might increase the density of the axial skeleton [8, 13, 28, 29]. The studies used in our review reported different percentages of patients with syndesmophytes varying between 7 and 55 % [7, 19, 27, 45], whereas some others excluded patients with syndesmophytes [22], and others did not address the issue of syndesmophytes at all [24, 26]. The presence of syndesmophytes could have falsely increased the BMD of lumbar spine in these studies, but we found comparable prevalence numbers of low BMD in the hip and spine, so probably the relatively low numbers of syndesmophytes in this early group did not influence the data considerably. But if it did, the real BMD would have been even lower.

Considering the high prevalence of decreased BMD in patients with early AS, the question arises whether or not patients should be treated for this condition. The answer to this question depends on the clinical relevance of this observation. Low BMD becomes clinically relevant when it leads to an increased risk of vertebral and nonvertebral fractures since these fractures are a serious cause of morbidity and reduced quality of life [62–64]. The link between decreased BMD and fracture risk is well known for the general healthy population [65]. In later stages of AS, the risk of vertebral fractures is also significantly increased, and low BMD is an important risk factor for that complication [9, 13–17, 33, 46]. Unfortunately, there is still no consensus whether or not decreased BMD in patients with early AS causes an increased fracture risk [9]. Very few and small studies have been performed in early AS. Jun et al. and van der Weijden et al. found a positive correlation between low BMD and vertebral fractures [25, 66]. Jun et al. found a relation between fractures and femoral BMD, whereas van der Weijden et al. found a relation with BMD of the lumbar spine. Nevertheless, other studies found no correlation with low BMD at all [10, 13].

## Conclusion

These studies show a high prevalence of low BMD (between 51 and 54 %) and a prevalence of 13–16 % of osteoporosis in AS patients within 10 years after diagnosis. This high prevalence was not to be expected in a relatively young and predominantly male population. No unambiguous correlation between decreased BMD and vertebral fracture risk could be demonstrated; therefore, further research is needed to determine the clinical relevance of low BMD in early AS patients by investigating the relation with vertebral and nonvertebral fractures in larger studies.

## Abbreviations

AS: Ankylosing spondylitis
BMD: Bone mineral density
DMARDs: Disease-modifying antirheumatic drugs
DXA: Dual x-ray absorptiometry
NSAIDs: Nonsteroidal anti-inflammatory drugs
SD: Standard deviation
SpA: Spondylarthropathy
WHO: World Health Organization
